# Effectiveness of digital healthcare in managing menstrual symptoms: a systematic review

**DOI:** 10.4069/whn.2025.05.22

**Published:** 2025-06-30

**Authors:** Mi Seon Seo, Bok-Nam Seo

**Affiliations:** 1 Department of Nursing, Jungwon University, Goesan, Korea; 2 Korea Institute of Oriental Medicine, Daejeon, Korea

**Keywords:** Digital health, Dysmenorrhea, Premenstrual syndromes, Systematic review, Women’s health

## Abstract

**Purpose:**

Pain and symptoms associated with dysmenorrhea and premenstrual syndrome (PMS) significantly impact the quality of life of women of reproductive age, potentially leading to various physical and psychological health problems that negatively influence daily activities. This systematic review aimed to evaluate the effectiveness of digital healthcare interventions in reducing health issues related to dysmenorrhea and PMS in women of reproductive age.

**Methods:**

We conducted a comprehensive systematic literature search of three databases (PubMed, Web of Science, and the Cochrane Library) up to August 2024 to identify randomized controlled trials evaluating digital health interventions for managing dysmenorrhea and PMS symptoms in reproductive-age women. Of the 750 articles initially identified, eight studies fulfilled the inclusion criteria. Two independent reviewers assessed the methodological quality of the included studies using the Cochrane Risk of Bias tool.

**Results:**

Eight studies were included, categorized as smartphone application-based interventions (n=5) and web-based programs (n=3). Digital healthcare interventions effectively reduced dysmenorrhea-related pain and positively impacted symptom management for both dysmenorrhea and PMS. PMS symptom severity was the most frequently measured outcome used to evaluate intervention effectiveness. These findings emphasize the potential of mobile and web-based platforms as accessible and effective tools for managing menstrual symptoms among women.

**Conclusion:**

This review highlights the importance of digital healthcare in reducing health issues related to menstrual symptom management. It also highlights the critical role of nursing in integrating digital healthcare solutions to support menstrual health management among reproductive-age women.

## Introduction

Dysmenorrhea and premenstrual syndrome (PMS) are among the most common health issues affecting women of reproductive age. Worldwide, over 50% of menstruating women and approximately 90% of adolescent girls experience dysmenorrhea, with 10% to 20% reporting it as acute, severe, or distressing. A recent systematic review and meta-analysis indicated that the prevalence of primary dysmenorrhea ranges from 60% to 73% [1,2]. Dysmenorrhea is characterized by cramping pain in the lower abdomen during menstruation and results from increased levels of prostaglandins in the uterus, leading to enhanced uterine contractions and subsequent pain [3]. Conversely, PMS involves various physical, emotional, and behavioral symptoms occurring during the luteal phase of the menstrual cycle and is associated with cyclical fluctuations in estrogen and progesterone, influencing neurotransmitters such as serotonin [4]. PMS affects approximately 47.8% of women of reproductive age worldwide [5]. Physical symptoms (e.g., abdominal bloating, breast tenderness, headaches, and limb swelling) and psychological symptoms (e.g., irritability, anxiety, confusion, and depression) typically manifest during the luteal phase, preceding menstruation, and subside once menstruation begins [6]. The physical and psychological symptoms associated with dysmenorrhea and PMS significantly impair the overall quality of life, negatively influencing relationships, academic performance, occupational activities, social interactions, and daily functioning [7].

Given the potentially debilitating nature of these symptoms, treatment approaches for dysmenorrhea have evolved, incorporating both pharmacological and non-pharmacological interventions. Standard treatments for primary dysmenorrhea typically involve nonsteroidal anti-inflammatory drugs and hormonal contraceptives [8]. Although effective due to their anti-prostaglandin properties, these medications may cause side effects, including gastrointestinal discomfort, cardiovascular complications, and mild neurological symptoms [9]. Similarly, selective serotonin reuptake inhibitors are commonly prescribed to manage PMS and premenstrual dysphoric disorder symptoms [10]; however, these medications frequently cause adverse effects such as nausea, drowsiness, and fatigue [11]. Non-pharmacological interventions, including yoga, acupuncture, and dietary supplements (such as vitamins and minerals), have demonstrated some effectiveness in reducing pain, but existing evidence supporting their efficacy remains limited [12,13].

Recently, digital healthcare, utilizing e-learning, telemedicine, and mobile technologies, has garnered increasing attention for its potential to enhance health outcomes in a cost-effective manner [14]. A major advantage of digital healthcare interventions is their ability to manage health concerns without constraints related to time or location. These interventions often monitor physical activity, heart rate, sleep patterns, and other health indicators using mobile or wearable devices [15]. Telemedicine, defined as delivering healthcare services via digital communication technologies, has gained recognition for improving treatment outcomes by providing expert consultation remotely, especially evident during the coronavirus disease 2019 pandemic [16]. Additionally, patient education and self-care coaching through mobile messaging programs have been widely adopted, demonstrated by daily management programs for couples facing infertility [17] and lifestyle improvement initiatives focused on prenatal health management during pregnancy [18]. Although various menstrual-related mobile applications incorporate pain and symptom management features, their overall effectiveness in improving health outcomes remains limited due to insufficient evidence-based content and suboptimal intervention designs, which fail to generate substantial user impact [19-24]. Other web-based programs have specifically targeted menstrual symptom management [25-27]. While these studies highlight the need for appropriate digital interventions addressing menstrual symptoms and improving overall health among reproductive-age women, specific findings are fragmented and inconsistent, complicating the clear identification of digital health interventions’ precise impacts. Nonetheless, existing studies indicate potential benefits in addressing critical gaps in healthcare services, including enhancing menstrual health knowledge and promoting overall well-being. A thorough review of current digital healthcare applications could substantially address these gaps, particularly by advancing menstrual health education and promoting overall well-being.

Thus, the objective of this study was to systematically review the types and effectiveness of digital healthcare interventions aimed at managing menstrual symptoms among women of reproductive age, evaluate their potential to improve women’s quality of life and explore strategies for their integration into daily life to enhance health outcomes. Additionally, this review aimed to provide essential evidence to guide the development of a comprehensive digital platform and evaluate its effectiveness in future research.

## Methods

Ethics statement: This study is a literature review of previously published studies; therefore, it was exempt from Institutional Review Board approval.

### Study design

The study protocol was registered with the International Prospective Register of Systematic Reviews (PROSPERO registration; CRD42024626233). Studies evaluating the types and effectiveness of digital healthcare interventions for managing women’s menstrual symptoms were identified and reviewed following the Preferred Reporting Items for Systematic Reviews and Meta-Analyses (PRISMA) 2020 guidelines of the Cochrane Collaboration [28].

### Inclusion and exclusion criteria

The core research question, “Is digital healthcare effective for managing menstrual symptoms in women?” was assessed using the PICO framework as follows:

(1) Participants: Women of reproductive age who experienced menstrual symptoms such as dysmenorrhea or PMS.

(2) Intervention: Digital healthcare interventions to manage menstrual symptoms.

(3) Comparison: Non-digital healthcare interventions for menstrual symptom management or a waitlist control group.

(4) Outcome: Indicators of menstrual symptom severity.

(5) Study design: Randomized controlled trials (RCTs).

The exclusion criteria were: i) non-human studies ii); non-experimental studies, such as descriptive surveys, review articles, and research reports; and iii) articles in languages other than English.

### Search strategy

Two researchers independently conducted a comprehensive literature search between December 18 and 31, 2024, using the international databases PubMed, Web of Science, and the Cochrane Library. There were no restrictions on the publication date; all studies published up to August 2024 were considered for analysis. The search strategy included controlled vocabulary (MeSH terms, Emtree), free-text keywords, and search operators (“AND,” “OR”). Researchers employed the following keyword combinations for the database searches: (“Dysmenorrhea” [MeSH terms] OR “Dysmenorrhea” OR “Menstruation Disturbances” [MeSH terms] OR “Menstruation Disturbances” OR “Menstrual Disorder” OR “Pelvic Pain” OR “Painful Menstruation” OR “Menstrual Pain” OR “Menstrual” OR “Menstruation” OR “premenstrual syndrome” [MeSH terms] OR “premenstrual syndrome”) AND (“digital health” [MeSH terms] OR “digital health” OR “telemedicine” [MeSH terms] OR “telemedicine” OR “e health” OR “electronic health” OR “m health” OR “mobile health” OR “mobile application” OR “computer-based” OR “APP”). Additionally, references from selected articles were manually reviewed to ensure no relevant studies were omitted. Further details regarding the search strategy are provided in Supplementary Table 1.

### Selection process and data extraction

The literature selection was conducted independently by two researchers from January 1 to 6, 2025. Selected studies were reviewed against predefined eligibility criteria. Disagreements during the selection process were resolved through consensus after re-examining the studies.

**Figure 1.**
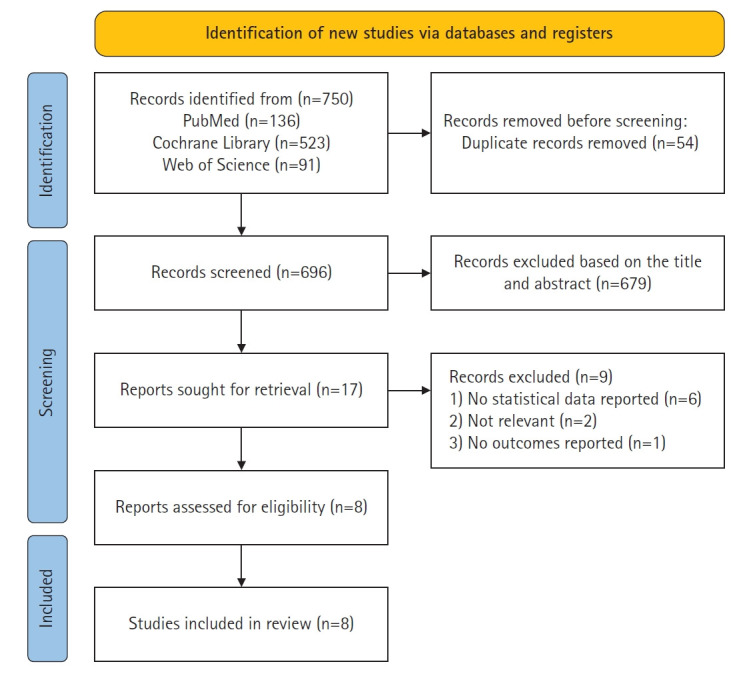
PRISMA 2020 flow diagram of the selection process.

The selection process consisted of identifying and removing duplicate articles using the reference management software EndNote 20 (Clarivate, Philadelphia, PA, USA), screening titles and abstracts for eligibility according to inclusion criteria, and performing full-text reviews for final analysis. Initially, 750 articles were identified using primary keywords. After eliminating duplicates (n=54), the titles and abstracts of 696 articles were screened, resulting in the selection of 17 articles. Upon full-text review, studies without statistical results (n=6), unrelated to digital healthcare for managing women’s menstrual symptoms (n=2), or lacking outcome reporting (n=1) were excluded. Ultimately, eight articles [20-27] were included in the systematic review (Figure 1).

### Quality assessment

Two researchers independently assessed the methodological quality of the eight selected studies, resolving disagreements through discussion. The quality of the included RCTs was evaluated using Cochrane’s Risk of Bias (RoB) 2.0 tool [29], which addresses five domains: (1) bias arising from the randomization process; (2) bias due to deviations from intended interventions; (3) bias due to missing outcome data; (4) bias in the measurement of the outcome; and (5) bias in the selection of reported results. Based on these assessments, each study was classified as having a “low risk of bias,” “some concerns,” or “high risk of bias.”

### Data extraction and analysis

Following study selection, data extraction was performed using a standardized form. Extracted data included general study characteristics (authors, publication year, study design), participant information (sample sizes for experimental and control groups, ages), intervention details (type, method, duration), outcome variables, and study findings. All extracted data were collaboratively reviewed and analyzed by the two researchers through detailed discussions.

## Results

### Study characteristics

The characteristics of the selected studies are outlined in Table 1. Of the eight studies included, three (37.5%) were published in 2024. Regarding the experimental intervention group sample sizes, three studies (37.5%) had fewer than 50 participants, two studies (25.0%) included between 50 and 100 participants, and three studies (37.5%) had more than 100 participants.

### Quality assessment

**Figure 2.**
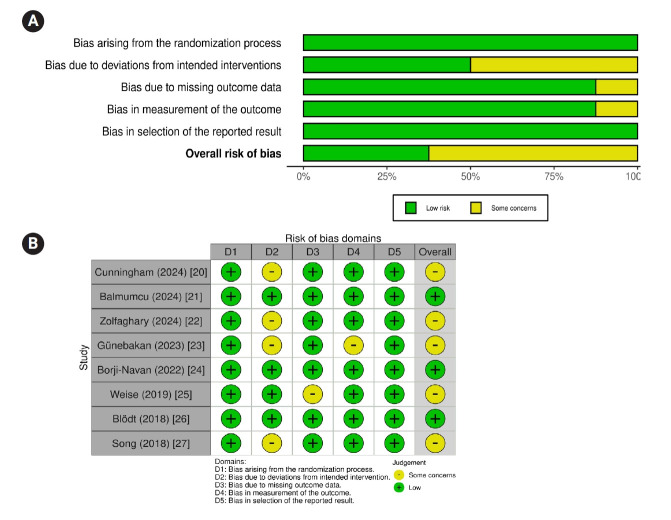
Risk of bias assessment in included studies. (A) Overall risk of bias graph. (B) Risk of bias summary.

Figure 2 presents the quality assessment of the eight studies evaluated using the Cochrane RoB 2.0 tool. Specifically, all studies (n=8) demonstrated a low risk of bias arising from the randomization process. For the domain related to deviations from intended interventions, four studies [20,22,23,27] showed a moderate risk, while the remaining four [21,24-26] were assessed as having a low risk. Regarding missing outcome data, one study [25] presented a moderate risk, whereas seven studies [20-24,26,27] were categorized as low risk. Bias in outcome measurement was moderate in one study [23] and low in the other seven studies [20-22,24-27]. All eight studies had a low risk of bias regarding the selection of reported results. Overall, five studies [20,22,23,25,27] exhibited a moderate risk of bias, and three studies [21,24,26] demonstrated a low risk of bias. However, due to the nature of the interventions, blinding of participants and researchers was a common concern, indicating a potential risk of bias across all studies.

### Effectiveness of digital healthcare interventions

A summary of the selected studies is presented in Table 2. Five studies utilized smartphone application-based interventions, which included a menstrual cycle tracking program with chatbot counseling [20], an online Pilates exercise program [21], a video-based yoga exercise program [23], a self-acupressure program [26], and a menstrual health management program [27]. Three studies applied web-based interventions comprising cognitive-behavioral therapy (CBT) [24,25] and stress prevention training [22]. Regarding intervention durations, four studies (50.0%) implemented interventions for two menstrual cycles [21,24-26], three studies (37.5%) for three menstrual cycles [20,23,27], and one study (12.5%) for six menstrual cycles [22].

The effectiveness of these interventions was evaluated using various measurement tools. The Premenstrual Symptoms Screening Tool was used in two studies (25.0%), while each of the following measurement instruments was utilized in only one study: Numeric Rating Scale, Incidence of Dysmenorrhea, Premenstrual Symptoms Screening Scale, Daily Record of Severity of Problems, Premenstrual Syndrome-Impact, and Menstrual Symptom Scale.

Six of the eight studies [20-25] specifically assessed the severity of PMS symptoms, making PMS symptom severity the most commonly measured outcome variable in evaluating digital healthcare interventions. Additionally, dysmenorrhea severity [26] and the incidence of dysmenorrhea [27] were each measured in one study. All eight studies reported positive effects of digital healthcare interventions on the management of menstrual symptoms (Table 2).

## Discussion

This systematic review evaluated the effectiveness of digital healthcare interventions for managing menstrual symptoms in women through an analysis of eight RCTs published between 2018 and 2024.

Previous research has shown that digital health solutions for women’s health have made significant progress. Digital healthcare interventions hold promise for effectively addressing key barriers to healthcare access by reducing time and space constraints as well as cost burdens. This convenience-oriented approach is particularly notable because it can offer tangible benefits in overcoming obstacles to accessing healthcare. Most women of reproductive age do not receive sufficient basic health knowledge about their menstrual cycle [30]. However, providing educational content related to reproductive health—such as menstruation, contraception, and pregnancy—through mobile applications has been shown to enhance reproductive health literacy [31] and empower women to manage their physical and mental well-being. The menstrual cycle is a critical health indicator for women, and the use of applications to monitor menstrual cycles and symptoms has been considered effective for promoting self-management. Despite menstruation being a natural biological process, it is often difficult for women to discuss it openly due to fear of stigma [32]. However, chatbots or online communities, which provide anonymity, facilitate open communication, resulting in high user satisfaction and positively influencing women’s health management.

In addition to digital healthcare interventions, lifestyle modifications such as physical activity have been explored as effective strategies for managing women’s health concerns, including PMS. Regular physical activity has been reported to decrease serum aldosterone levels and increase estrogen and progesterone levels, thereby alleviating PMS symptoms [33]. Application-based exercise programs are particularly promising due to their flexibility regarding time and location, as well as their capacity to be personalized to users’ preferences and busy schedules. However, the application-based interventions identified in this review were limited to specific exercise types such as yoga or Pilates. Although these modalities have demonstrated effectiveness, the limited variety of exercise options might hinder user engagement and adherence, particularly among individuals with different preferences or physical capabilities. Therefore, developing programs that incorporate diverse exercise forms, such as stretching, aerobic exercises, and strength training, may enhance user satisfaction and support sustained adherence.

In addition to physical activity, addressing psychological factors is recognized as an essential component in managing menstrual symptoms. Web-based interventions, including CBT [24,25] and stress prevention training [22], have been implemented to target psychological issues such as depression, anxiety, and stress, which are known to influence PMS severity [34]. Consequently, providing women experiencing PMS with web-based programs designed to help them choose coping strategies that enhance positive emotions and manage stress has effectively reduced functional and psychological impairments, improving PMS symptoms. Compared with traditional CBT, this web-based approach is positively regarded due to its cost-effectiveness, geographic and temporal accessibility, and provision of a safe space for individuals uncomfortable with face-to-face therapy. Enhancing these benefits by integrating personalized features—such as mood tracking, adaptive content delivery, and menstrual cycle-based symptom prediction—may further improve the effectiveness and user engagement of web-based interventions for PMS.

The main strength of this systematic review is its emphasis on high-quality evidence derived exclusively from RCTs, with a rigorous risk-of-bias assessment conducted using the RoB 2.0 tool. Nevertheless, this review has several limitations. First, the variability introduced by different assessment tools across studies may have influenced the consistency of results. Second, the generalizability of findings may be constrained if sample sizes, particularly in intervention and control groups, are insufficiently large. Additionally, only three of the eight included studies were assessed as having a low risk of bias, potentially affecting the overall strength and interpretability of the evidence presented.

In conclusion, this review emphasizes that digital healthcare interventions represent effective and cost-efficient strategies for alleviating symptoms of dysmenorrhea and PMS. These interventions not only help reduce physical and emotional symptoms—such as pain, anxiety, and depression—but are also recognized as valuable self-management tools that can complement existing treatment modalities. However, existing approaches have predominantly focused on individual components of menstrual symptom management rather than addressing menstrual health comprehensively through integrated approaches. To enhance the effectiveness of digital healthcare interventions, it is recommended that these tools be consistently incorporated into daily routines and health-related behaviors.

The findings of this study can inform the development of personalized digital health platforms for managing women’s health, which collect data on menstrual cycles, menstruation-related symptoms, and lifestyle factors. These data could then be leveraged to deliver individualized education, physical activity programs, and emotional regulation strategies tailored specifically to each woman’s unique needs and conditions.

## Figures and Tables

**Table 1.** General characteristics of the included studies (N=8)

Variable	Categories	n (%)	Reference
Publication year	2024	3 (37.5)	[20], [21], [22]
	2023	1 (12.5)	[23]
	2022	1 (12.5)	[24]
	2019	1 (12.5)	[25]
	2018	2 (25.0)	[26], [27]
Sample size	<50	3 (37.5)	[21], [23], [24]
	50–100	2 (25.0)	[22], [25]
	>100	3 (37.5)	[20], [26], [27]

**Table 2.** Summary of the included studies, in chronological order (N=8)

First author (year) [Ref]	Country	Eligibility	Age range (year)	Intervention type (intervention period)	Intervention content	Group	Outcome variable	Assessment tools	Main results
						Experimental/Control			
Cunningham (2024) [20]	USA	≥18 years old who reported moderate-to-severe PMS or PMDD symptoms	18–54	Digital health app (3 month)	Flo app (menstrual cycle and symptom tracking, health education, interactive community)	194/244	Menstrual Health literacy	PSST	Significant improvements in health literacy, menstrual awareness, well-being, and PMS/PMDD symptom burden (*p*<.001)
							Menstrual health awareness	WHO Quality of Life-BREF	
							PMS/PMDD symptom burden	Q-LES-Q	
							General health and well-being		
Balmumcu (2024) [21]	Türkiye	Regular menstrual cycles and diagnosed with PMS via PMSS (score >110); BMI between 18.5 and 24.9 kg/m²	18–24	Digital health app (8 wks)	Physical activity+mHealth (WhatsApp-based)	34/34	PMS symptom severity	PMSS	Significant reduction in total PMSS scores at 8 weeks compared to the control group (*p*<.001)
							Subscales: depressive affect, anxiety, irritability, fatigue, sleep/appetite changes, pain		
Zolfaghary (2024) [22]	Iran	Regular menstrual cycles	18–38	Web-based programs (6 wks)	SIT counseling intervention: online modules covering relaxation, coping skills and stress management techniques	50/50	PMS symptom severity	PSST	Significant reduction in PMS symptoms PSST, HADS, PSS-14 and SDS immediately after and up to two menstrual cycles post-intervention (*p*<.001)
		Diagnosed with PMS via PSST					Anxiety and depression	HADS	
		Score of 8 or higher on the HADS					Perceived stress	PSS-14	
							Disability in functioning	SDS	
							Psychological well-being		
Günebakan (2023) [23]	Türkiye	Healthy premenopausal women	18–45	Digital health app (12 wks)	Tele-yoga training	16/16	Menstruation Symptoms	MSS	Significant improvements in MSS (52.31 vs. 67.19), NHP (sleep, energy, emotion, social isolation), and BDS *p*<.05)
		Had never practiced yoga before			- 2×/wk, 45-min Zoom session		Health-related quality of life	BDS	
					- Included asanas, pranayama (breathing), savasana (relaxation)		Depression and anxiety	State and Trait Anxiety Inventory	
							Self-esteem Awareness	NHP	
Borji-Navan (2022) [24]	Iran	Women university students who had moderate-to-severe PMS	18–35	Web-based programs (8 wks)	Internet-based CBT: cognitive restructuring, education, lifestyle (diet/exercise), coping skills, delivered weekly via LMS with SMS/telegram reminders	46/46	PMS symptom severity	Daily Record of Severity of Problems	Significantly lower total PMS symptoms 10.4 vs. 20.2 (*p*<.001), perimenstrual quality of life 64.2 vs. 50.3 (*p*<.001)
		Have regular menstrual cycles (25–35 days) for at least 6 mo					Quality of life	Q-LES-Q	
							PMS-related disability	SDS	
							Menstrual attitudes	Menstrual Attitude Questionnaire	
Weise (2019) [25]	Germany	DSM-5 confirmed PMDD	18–45	Web-based programs (8 wks)	Therapist-guided internet-based CBT: 14 online modules	86/88	Functional and psychological impairment	Premenstrual Impact Questionnaire	Significant improvements FI from 25.00 → 17.09 (*p*<.001), PI from 28.69 → 20.68 (*p*<.001), SI from 22.98 → 9.32 (*p*<.001), PDI from 32.49 → 15.54 (*p*<.001)
		Regular menstrual cycles (24–31 days)			Includes CBT techniques and lifestyle changes		Symptom intensity	Perceived Stress Scale	
							Symptom disability	Prospective Symptom Diary	
							Impact on everyday life	PDI	
Blödt (2018) [26]	Germany	Self-reported moderate-to-severe (NRS≥6)	18–34	Digital health app (6 menstrual cycles)	AKUD app	111/110	Pain intensity	NRS	Significant reduction in pain (–0.6 NRS at cycle 3, –1.4 NRS at cycle 6, *p*<.001), lower medication use (OR, 0.3–0.5; *p*<.001)
		Regular menstrual cycles			: App-based self-acupressure instructions (video), stimulation of SP6, LI4, LR3 points; 1-min per point, 2×/day from 5 days		Number of pain days	Self-reported app-based diary and questionnaire entries	
							Pain medication use		
							Sick leave days		
							Self-efficacy expectation		
Song (2018) [27]	Japan	Female full-time workers	20–45	Digital health app (3 month)	Karada-no-kimochi app (Tracks menstrual cycle and symptoms; provides lifestyle tips)	612/914	Incidence of depression, dysmenorrhea, PMS	Online survey for diagnosis visits	Significant reduction in depression (0.16% vs. 0.77%, *p*=.035) and dysmenorrhea (0.33% vs. 1.31%, *p*=.013) in the intervention group
		Regular menstrual cycles					Absenteeism and presenteeism	Measured using the Work Productivity and Activity Impairment Questionnaire	Economic evaluation suggested cost savings of JPY 130,000 (USD 1,170) per individual
		Never used the “Karada-no-kimochi” app					Cost-effectiveness	Patient Health Questionnaire	

BMI: body mass index; BDS: Beck Depression Scale; CBT: cognitive behavioral therapy; DSM-5: Diagnostic and Statistical Manual of Mental Disorders, Fifth Edition; FI: functional impairment; HADS: Hospital Anxiety and Depression Scale; JPY: Japanese yen; LMS: learning management system; MSS: Menstrual Symptom Scale; NHP: Nottingham Health Profile; NRS: Numeric Rating Scale; OR: odds ratio; PDI: Pain Disability Index; PI: psychological impairment; PMDD: premenstrual dysphoric disorder; PMSS: Premenstrual Syndrome Scale; PMS: premenstrual syndrome; PSST: Premenstrual Screening Tool; PSS-14: Perceived Stress Scale, 14-item version; Q-LES-Q: Quality of Life Enjoyment and Satisfaction Questionnaire; SDS: Sheehan Disability Scale; SI: symptom intensity; SIT: stress inoculation training; SMS: short message service; USD: United States dollar; WHO Quality of Life-BREF: World Health Organization Quality of Life Instrument, abbreviated version

## Supplementary materials

Further details on supplementary materials are presented online (available at https://doi.org/10.4069/whn.2025.05.22).

Supplementary Table 1. Search strategies
