# Supplementary material for: Effectiveness of digital healthcare in managing menstrual symptoms: a systematic review
Source: Womens Health Nurs. 2025 Jun 30;31(2):108–18. doi: 10.4069/whn.2025.05.22 (PMC12245543; doi:10.4069/whn.2025.05.22)
Supplement: Supplementary Table 1. — Search strategies [file whn-2025-05-22-Supplementary-Table-1.pdf]

Supplementary Table 1. Search strategies

Search database	Search terms	Search period	Search results
PubMed	#1"Dysmenorrhea" [MeSH Terms] OR "Dysmenorrhea" [Title/Abstract] OR "Menstruation Disturbances" [MeSH Terms] OR "Menstruation Disturbances" [Title/Abstract] OR "Menstrual Disorder" [Title/Abstract] OR "Painful Menstruation" [Title/Abstract] OR "Menstrual Pain" [Title/Abstract] OR "Menstruation" [Title/Abstract] OR "premenstrual syndrome" [MeSH Terms] OR "premenstrual syndrome"[Title/Abstract]	2024-12-18 ~ 2024/12/31	46,681
	#2 "digital health"[MeSH Terms] OR "digital health"[Title/Abstract] OR "telemedicine"[MeSH Terms] OR "telemedicine"[Title/Abstract] OR "e health"[Title/Abstract] OR "electronic health"[Title/Abstract] OR "m health"[Title/Abstract] OR "mobile health"[Title/Abstract] OR "mobile application"[Title/Abstract] OR "computer-based" [Title/Abstract] OR "APP"[Title/Abstract]		177,059
	#1 AND #2		136
	("Dysmenorrhea" [MeSH Terms] OR "Dysmenorrhea" [Title/Abstract] OR "Menstruation Disturbances" [MeSH Terms] OR "Menstruation Disturbances" [Title/Abstract] OR "Menstrual Disorder" [Title/Abstract] OR "Painful Menstruation" [Title/Abstract] OR "Menstrual Pain" [Title/Abstract] OR "Menstruation" [Title/Abstract] OR "premenstrual syndrome[MeSH Terms] OR "premenstrual syndrome"[Title/Abstract]") AND ("digital health"[MeSH Terms] OR "digital health"[Title/Abstract] OR "telemedicine"[MeSH Terms] OR "telemedicine"[Title/Abstract] OR "e health"[Title/Abstract] OR "electronic health"[Title/Abstract] OR "m health"[Title/Abstract] OR "mobile health"[Title/Abstract] OR "mobile application"[Title/Abstract] OR "computer-based" [Title/Abstract] OR "APP"[Title/Abstract])		
Cochrane Library	#1"Dysmenorrhea" [MeSH Terms] OR "Dysmenorrhea" [Title/Abstract] OR "Menstruation Disturbances" [MeSH Terms] OR "Menstruation Disturbances" [Title/Abstract] OR "Menstrual Disorder" [Title/Abstract] OR "Painful Menstruation" [Title/Abstract] OR "Menstrual Pain" [Title/Abstract] OR "Menstruation" [Title/Abstract] OR "premenstrual syndrome" [MeSH Terms] OR "premenstrual syndrome" [Title/Abstract]	2024-12-18 ~ 2024/12/31	10,642
	#2 "digital health"[MeSH Terms] OR "digital health"[tiab] OR "telemedicine"[MeSH Terms] OR "telemedicine"[tiab] OR "e health"[tiab] OR "electronic health"[tiab] OR "m health"[tiab] OR "mobile health"[tiab] OR "mobile application"[tiab] OR "computer-based" [tiab] OR "APP"[tiab]		102,121
	#1 AND #2		523
Web of Science	#1 TS= ("Dysmenorrhea" OR "Menstruation Disturbances" OR "Menstrual Disorder" OR "Painful Menstruation" OR "Menstrual Pain" OR "Menstruation" OR "premenstrual syndrome")	2024-12-18 ~ 2024/12/31	11,743
	#2 TS= ("digital health" OR "telemedicine" OR "e health" OR "electronic health" OR "m health" OR "mobile health" OR "mobile application" OR "computer-based" OR "APP")		143,906
	#1 AND #2		91
